# Associations of maternal pre-pregnancy BMI and gestational weight gain with the risks of adverse pregnancy outcomes in Chinese women with gestational diabetes mellitus

**DOI:** 10.1186/s12884-023-05657-8

**Published:** 2023-06-03

**Authors:** Jiang-Feng Ke, Sheng Liu, Ri-Le Ge, Li Ma, Mei-Fang Li

**Affiliations:** 1 Department of Radiation Oncology, Clinical Oncology School of Fujian Medical University, Fujian Cancer Hospital, Fuzhou, China; 2 Department of Endocrinology & Metabolism, Shanghai Clinical Medical Center of Diabetes, Shanghai Key Clinical Center of Metabolic Diseases, Shanghai Institute for Diabetes, Shanghai Key Laboratory of Diabetes, Shanghai Sixth People’s Hospital Affliated to Shanghai Jiao Tong University School of Medicine, Shanghai, China; 3 Department of Emergency, Shanghai Sixth People’s Hospital Affliated to Shanghai Jiao Tong University School of Medicine, Shanghai, China; 4 Shanghai Medical Emergency Center, Shanghai, China; 5 Department of General Practice, Shanghai Sixth People’s Hospital Affiliated to Shanghai Jiao Tong University School of Medicine, Shanghai, China; 6 Department of Obstetrics and Gynecology, Shanghai Clinical Center for Severe Maternal Rescue, Shanghai Sixth People’s Hospital Affiliated to Shanghai Jiao Tong University School of Medicine, Shanghai, China

**Keywords:** Pre-pregnancy overweight/obesity, Gestational weight gain, Gestational diabetes mellitus, Adverse pregnancy outcomes

## Abstract

**Background:**

Give the high background risk of adverse pregnancy outcomes (APOs), it is important to understand the associations of maternal pre-pregnancy body mass index (ppBMI), gestational weight gain (GWG) with APOs in women with gestational diabetes mellitus (GDM). We addressed the independent and joint associations of maternal ppBMI and GWG with APOs in Chinese women with GDM.

**Methods:**

764 GDM women with singleton delivery were studied and they were stratified into three weight groups by ppBMI (underweight, normal weight and overweight/obesity) following classification standards for Chinese adults and three GWG groups (inadequate, adequate, excessive GWG) by the 2009 Institute of Medicine guidelines, respectively. Univariate and multivariate logistic regression analyses were performed to estimate the odds ratios of APOs.

**Results:**

Maternal overweight/obesity was associated with increased odds of pregnancy-induced hypertension [PIH, adjusted odds ratio (aOR): 2.828, 95% confidence interval (CI) 1.382–5.787], cesarean delivery (CS) (aOR 2.466, 95%CI 1.694–3.590), preterm delivery (aOR 2.466, 95%CI 1.233–4.854), LGA (aOR 1.664, 95%CI 1.120–2.472), macrosomia (aOR 2.682, 95%CI 1.511–4.760) and any pregnancy complication (aOR 2.766, 95%CI 1.840–4.158) compared with healthy weight. Inadequate GWG was less likely to develop PIH (aOR 0.215, 95%CI 0.055–0.835), CS (aOR 0.612, 95%CI 0.421–0.889) and any pregnancy complication (aOR 0.628, 95%CI 0.435–0.907), but had higher risk of preterm birth (aOR 2.261, 95%CI 1.089–4.692), while excessive GWG was more vulnerable to LGA (aOR 1.929, 95%CI 1.272–2.923), macrosomia (aOR 2.753, 95%CI 1.519–4.989) and any pregnancy complication (aOR 1.548, 95%CI 1.006–2.382) as compared to adequate GWG. Furthermore, compared to normal weight mothers with adequate GWG, obese mothers with excessive GWG had the highest risk of any pregnancy complication (aOR 3.064, 95%CI 1.636–5.739).

**Conclusions:**

Maternal overweight/obesity and GWG were associated with APOs in the already high-risk settings of GDM. Obese mothers with excessive GWG may confer the greatest risk of adverse outcomes. It was very helpful to reduce the burden of APOs and benefit GDM women by promoting a healthy pre-pregnancy BMI and GWG.

## Background

The prevalence of overweight and obesity for women at childbearing age has increased dramatically and globally. Pre-pregnancy overweight/obesity is garnering more attention as a determinant of pregnancy outcomes for both maternal and child wellbeing. For example, the global incidence rate of overweight/obese women rose from 29.8% to 1980 to 38.0% in 2013 [1]. In China, overweight/obese is common in 10–24% of pregnant women while almost 11–13% pregnant women were underweight [2, 3]. Accumulating evidences showed that maternal pre-pregnancy overweight/obesity increased the risks of adverse perinatal outcomes such as preterm birth, large-for-gestational age (LGA) and macrosomia, while maternal underweight was related to low birth weight and small-for-gestational age (SGA) in non-diabetic population [4–6]. However, few studies have been made on GDM women.

Next to maternal pre-pregnancy body mass index (ppBMI), too little or excessive gestational weight gain (GWG) during pregnancy could also generate widespread damages to mothers and infants [7, 8]. To optimize pregnancy outcomes, the Institute of Medicine (IOM) published the recommended guidelines on appropriate GWG first in 1990 and revised in 2009 [10, 11]. Despite the updated IOM’s GWG targets include a more specific and narrower range based on ppBMI and apply to women of all ethnicities and statures, heterogeneity of race and ethnicity does exist and there is still approximately 36% of pregnant women gained weight above the IOM’s guidelines [12–14]. For example, high rates of excessive GWG (EGWG) and pre-pregnancy overweight/obesity were reported in Europe (36% and 29%, respectively) and USA (44% and 59.5%, respectively) [15, 16], while these figures were lower in China (27.6% and 6.1%, respectively) [17, 18]. In addition, most of the current evidences displayed that inadequate and excessive GWG were related to poor pregnancy outcomes for non-diabetic women in Western or high-income countries [9, 19, 20], there is limited information regarding the impacts of the revised IOM’s GWG targets on pregnancy outcomes in Asian women with GDM.

The prevalence of GDM is growing in line with delayed motherhood, the rising incidence rate of overweight and obesity and unhealthy lifestyles. In Shanghai, 24.1% of pregnant women were identified as GDM in 2016 according to our early study of 3269 Chinese singleton pregnant women [21]. Obesity is often accompanied by GDM [22]. Fat, as an endocrine organ, interacts with abnormal glucose metabolism. Nevertheless, experimental studies found that exceeding a certain level of metabolic disorders resulting in GDM does not require increase in the adipose tissue mass [23–25]. Therefore, it is unclear whether obesity combined with excessive GWG could pose additional risk to women with GDM and whether obesity or excessive GWG alone could generate worse perinatal outcomes in women with GDM.

To address the above-mentioned questions, we made this retrospective and real-world study to explore the associations between maternal ppBMI, GWG and adverse pregnancy outcomes in Chinese GDM women who face a greater background risk of adverse outcomes. Furthermore, we evaluated the impacts of different GWG categories on the rates of adverse pregnancy outcomes in Chinese GDM women with different pre-pregnancy BMI categories.

## Materials and methods

### Subjects and study design

We conducted a retrospective cohort study using data from our previous studies [21, 26, 27]. Briefly, a total of 4178 pregnant women who performed an oral glucose tolerance test (OGTT) at 24–28 weeks and delivered in the obstetrics department of Shanghai Sixth People’s Hospital Affiliated to Shanghai Jiao Tong University School of Medicine from January to December in 2016 were continuously observed. Among them, 787 singleton pregnant women were diagnosed with GDM. We further excluded women if they met any of the criteria as follows:1) incomplete information on demographics, complications, delivery information; 2) reproductive system anatomy abnormalities, a history of chronic diseases or malignancy; 3) women with previous pregnancy complications. Finally, 764 GDM women joined in the current study.

**Fig. 1:**
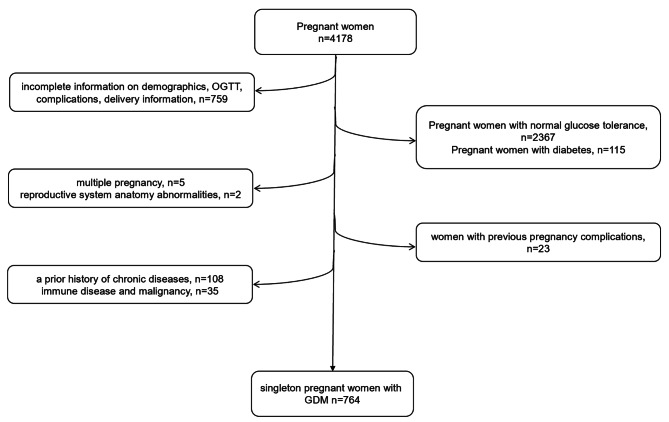
The flow chart of the subject enrollment in our study

Our study was approved by the ethics committee of Shanghai Sixth People’s Hospital Affiliated to Shanghai Jiao Tong University School of Medicine, and written informed consents were obtained from the whole participants following the Declaration of Helsinki. The flow chart of the subject enrollment in our study was shown in Fig. 1.

### Data collection and definitions

Maternal characteristics including maternal age, body weight and height before pregnancy, gestational age, primiparity, number of previous pregnancies and births, maternal weight at delivery, implementation on doctors’ advices, characteristics of their offspring including neonatal sex, birth weight and height, Apgar scores and adverse pregnancy outcomes were obtained from their pre-natal care visits and hospital admission/discharge records.

GDM was identified when fasting blood glucose (FBG) ≥ 5.1 mmol/L during the first trimester or any plasma glucose after a 75 g OGTT performed as: FBG ≥ 5.1 mmol/L, 1 h after glucose load ≥ 10.0 mmol/L or 2 h after glucose load ≥ 8.5 mmol/L using the criteria of International Association of Diabetes and Pregnancy Study Groups (IADPSG) [28]. For each GDM woman, doctors’ advices on lifestyle changes including diet modification, self-monitor glucose and exercise, and the use of insulin when lifestyle changes failed to maintain adequate glycemic targets which were well-described previously [29]. All data on whether they followed doctors’ advices and adopted insulin therapy were manually collected from their medical records on a fortnightly basis of pre-natal care visit. Among them, 545 subjects achieved more than 80% doctors’ advices who were classified as implementation on doctors’ advices, as described in our previous study [21].

PpBMI was obtained as pre-pregnancy weight (kg)/height(m)^2^, and participants in our study were categorized into three weight groups by ppBMI classification standards for Chinese adults: underweight (< 18.5 kg/m^2^), normal weight (18.5–23.9 kg/m^2^) and overweight/obesity (≥ 24 kg/m^2^) [30]. GWG was calculated by deducting pre-pregnancy weight from maternal at-delivery weight, and each woman was stratified into three GWG groups by the revised IOM’s guidelines as inadequate, adequate, or excessive. Adequacy of GWG was defined according to the revised IOM’s recommended GWG for underweight, normal weight, overweight and obese women who could add 12.5–18 kg, 11.5–16 kg, 7-11.5 kg, and 5–9 kg, respectively [11]. Inadequate GWG (iGWG) or excessive GWG (EGWG) was successively defined as below or above the IOM’s GWG targets in each ppBMI status. Moreover, given the relatively small sample size of underweight group, the associations of the various GWG ranges with pregnancy complications were only conducted in maternal normal and overweight/obese categories under the subgroup analysis, rather than maternal underweight group.

### Adverse pregnancy outcomes

The primary outcome in the present study was any pregnancy complication [including pregnancy-induced hypertension (PIH), preeclampsia, cesarean delivery (CS), preterm delivery, low birth weight, macrosomia, LGA, SGA) and the secondary outcome was PIH, preeclampsia, CS, preterm delivery, low birth weight, macrosomia, LGA and SGA, respectively.

PIH was defined as new-onset systolic blood pressure ≥ 140 mmHg and/or diastolic blood pressure ≥ 90 mmHg which occurred after 20-week gestation [31]. Preeclampsia was diagnosed as PIH accompanied by proteinuria 0.3 g/ 24 h [31]. CS was recorded by a midwife or doctor. Preterm delivery was defined as < 37 gestational weeks at birth [31]. Low birth weight was identified as birthweight < 2500 g and macrosomia was identified as birthweight ≥ 4000 g [31]. SGA and LGA were successively referred to birthweight < 10th and > 90th percentile for gestational age on a standard growth chart [32].

### Statistical analysis

All statistical analyses were made by SPSS 19.0. The measurement data of normal distribution were represented as mean ± standard deviation, and One-way ANOVA with LSD was applied to make comparisons among groups. Non-normal distribution was expressed as median (interquartile range), and the Kruskal-Wallis test was used to compare among groups. Enumeration data were described as frequency (percent) and the chi-squared test was adopted to make comparisons among groups. To assess the impacts of maternal ppBMI and GWG on adverse pregnancy outcomes in GDM women, multiple logistic regression models were used and odds ratios were obtained after adjustment for the following covariates that were the statistically significant features in Table 1 and had indeed proven influences on maternal and neonatal adverse outcomes reported by the previous literatures [21, 33]: maternal age, primiparity, gestational weeks, GWG, FBG and implementation on doctors’ advices. Models for neonatal outcomes were also adjusted for PIH and preeclampsia. *P* < 0.05 (two-sided) was regarded as statistically significant.

**Table 1:** Characteristics of maternal and their offsprings according to pre-pregnancy BMI and GWG status in GDM women

Variables	Underweight (n = 65)	Normal weight (n = 481)	Overweight/obese (n = 218)	*p* value			iGWG (n = 238)	Adequate GWG (n = 329)	EGWG (n = 197)	*p v*alue
*Maternal characteristics*										
Maternal age (years)^a^	29(26–31)	30(27–34)	31(28–35)	< 0.001			30(28–34)	30(28–34)	30(27–34)	0.292
Gestational age (weeks)	38.3 ± 4.2	38.6 ± 2.5	38.6 ± 2.3	0.717			37.9 ± 2.9	38.8 ± 1.7	39 ± 1.7	< 0.001
Pre-pregnancy BMI (kg/m^2^)^a^	17.88(17.45–18.17)	21.26(19.95–22.48)	25.79(24.83–27.94)	< 0.001			21.48(19.92–23.08)	21.88(19.88–24.22)	23.44(20.80-25.46)	< 0.001
Primiparity, n (%)	45(69.2)	304(63.2)	119(54.6)	0.028			139(58.4)	190(57.8)	139(70.6)	0.017
Number of previous pregnancies, n (%)				0.275						0.988
0 time	27(41.5)	200(41.6)	74(33.9)				93(39.1)	128(38.9)	80(40.6)	
1 time	22(33.8)	135(28.1)	69(31.7)				70(29.4)	97(29.5)	59(29.9)	
2 times and above	16(24.6)	146(30.4)	75(34.4)				75(31.5)	104(31.6)	58(29.4)	
Number of previous births, n (%)				0.164						0.027
0 time	45(69.2)	304(63.2)	119(54.6)				139(58.4)	190(57.8)	139(70.6)	
1 time	18(27.7)	167(34.7)	93(42.7)				92(38.7)	131(39.8)	55(27.9)	
2 times and above	2(3.1)	10(2.1)	6(2.8)				7(2.9)	8(2.4)	3(1.5)	
GWG (kg)^a^	13(10–16.25)	13(10–15.75)	10(7.5–14)	< 0.001			9(6–10)	13(11–15)	17(15–20)	< 0.001
75 g OGTT										
FBG (mmol/l)	4.67 ± 0.51	4.77 ± 0.52	4.90 ± 0.55	< 0.001			4.69 ± 0.55	4.78 ± 0.52	4.95 ± 0.51	< 0.001
1 h PBG (mmol/l)	8.51 ± 1.56	8.64 ± 1.56	8.57 ± 1.62	0.849			8.66 ± 1.58	8.57 ± 1.59	8.59 ± 1.56	0.845
2 h PBG (mmol/l)	7.44 ± 1.58	7.14 ± 1.61	7.19 ± 1.63	0.446			7.41 ± 1.72	7.05 ± 1.60	7.13 ± 1.46	0.055
Implementation on doctors’ advices, n (%)	47(72.3)	341(70.9)	157(72)	0.939			168(70.6)	239(72.6)	138(70.1)	0.779
*Newborn characteristics*										
Male, n (%)	32(49.2)	249(51.8)	117(53.7)	0.760			117(49.2)	170(51.7)	111(56.3)	0.434
Birth weight (g)	3157 ± 607	3347 ± 507	3432 ± 598	0.002			3189 ± 619	3347 ± 481	3564 ± 491	< 0.001
Birth height (cm)	49.24 ± 3.13	49.70 ± 2.23	49.66 ± 3.74	0.475			49.26 ± 3.29	49.63 ± 3.15	50.14 ± 0.88	0.006
Apgar scores	9.63 ± 0.77	9.87 ± 0.93	9.79 ± 0.77	0.227			9.67 ± 1.02	9.85 ± 1.02	9.99 ± 0.97	0.005

Abbreviations: BMI: pre-pregnancy body mass index; GWG: gestational weight gain; GDM: gestational diabetes mellitus; iGWG: inadequate GWG; EGWG: excessive GWG; OGTT, Oral glucose tolerance test; FBG, Fasting blood glucose; 1 h PBG, 1 h Postprandial blood glucose; 2 h PBG, 2 h Postprandial blood glucose

Continuous variables were expressed as mean ± standard deviation or median with interquartile range, while categorical variables were expressed as percentages

^a^Non-normal distribution of continuous variables

## Results

### Demographic and clinical characteristics of the studied subjects

Of all 764 GDM women, the median maternal age, ppBMI and total GWG were 30 years (IQR 27–34), 22.03 kg/m^2^ (IQR 22.01–24.22) and 12 kg (IQR 10–15), respectively. The demographic and clinical characteristics of maternal and their offspring based on ppBMI and GWG statuses are presented in Table 1. According to the ppBMI status, 65 women (8.5%) were underweight, 481 (63%) were of normal weight, and 218 (28.5%) were overweight/obese. Compared with normal weight and underweight groups, maternal pre-pregnancy overweight/obese group has significantly higher maternal pre-pregnancy overweight/obese group has significantly higher maternal age, FBG, and neonatal birth weight and lower primiparity, as well as GWG (all *p* < 0.05). In addition, based on the revised IOM’s guidelines for GWG, 238 women (31.2%) exhibited iGWG, 329 (43.1%) exhibited adequate GWG, and 197 (25.7%) exhibited EGWG. Compared to women with adequate or inadequate GWG, those with EGWG exhibited remarkably higher gestational age, ppBMI, primiparity, FBG, neonatal birth weight and height as well as Apagar scores (all *p* < 0.05).

### Maternal ppBMI status and risks of adverse pregnancy outcomes in GDM women

Table 2 demonstrated that the effects of different maternal ppBMI status on adverse pregnancy complication. As compared to normal weight mothers, overweight/obesity mothers had 2.766-higher risk of any pregnancy complication even after multivariable adjustments. Mothers with overweight/obesity had also markedly increased risks for PIH [adjusted odds ratio (aOR) 2.828, 95% confidence interval (CI) 1.382–5.787], CS (aOR 2.466, 95%CI 1.694–3.590), preterm delivery (aOR 2.466, 95%CI 1.233–4.854), LGA (aOR 1.664, 95%CI 1.120–2.472) and macrosomia (aOR 2.682, 95%CI 1.511–4.760). However, no statistical significance was found on the risks of adverse pregnancy outcomes between normal weight mothers and underweight mothers.

**Table 2:** Maternal ppBMI status and risks of adverse pregnancy outcomes in GDM women

Outcomes	Underweight	Normal weight	Overweight/obese
PIH, n (%)	2(3.1)	19(4)	22(10.1)
Unadjusted OR (95%CI)	0.770(0.175–3.386)	1(ref)	2.723(1.442–5.145)*
Adjusted OR (95%CI)	0.971(0.211–4.467)	1(ref)	2.828(1.382–5.787)*
Preeclampsia, n (%)	1(1.5)	13(2.7)	10(4.6)
Unadjusted OR (95%CI)	0.563(0.072–4.372)	1(ref)	1.731(0.747–4.011)
Adjusted OR (95%CI)	0.667(0.081–5.468)	1(ref)	1.959(0.783–4.902)
CS, n (%)	23(35.4)	210(43.7)	131(60.1)
Unadjusted OR (95%CI)	0.707(0.412–1.212)	1(ref)	1.943(1.403–2.691)**
Adjusted OR (95%CI)	0.790(0.437–1.429)	1(ref)	2.466(1.694–3.590)**
Preterm delivery, n (%)	4(6.2)	25(5.2)	26(11.9)
Unadjusted OR (95%CI)	1.191(0.401–3.538)	1(ref)	2.459(1.385–4.367)*
Adjusted OR^a^ (95%CI)	0.682(0.148–3.139)	1(ref)	2.446(1.233–4.854)*
LGA, n (%)	11(16.9)	113(23.5)	70(32.1)
Unadjusted OR (95%CI)	0.663(0.335–1.312)	1(ref)	1.540(1.081–2.194)*
Adjusted OR^b^ (95%CI)	0.624(0.293–1.330)	1(ref)	1.664(1.120–2.472)*
SGA, n (%)	1(1.5)	7(1.5)	4(1.8)
Unadjusted OR (95%CI)	1.058(0.128–8.740)	1(ref)	1.266(0.367–4.369)
Adjusted OR^b^ (95%CI)	-	1(ref)	1.517(0.394–5.849)
Macrosomia, n (%)	1(1.5)	39(8.1)	32(14.7)
Unadjusted OR (95%CI)	0.178(0.024–1.317)	1(ref)	1.981(1.203–3.261)*
Adjusted OR^c^ (95%CI)	0.178(0.023–1.390)	1(ref)	2.682(1.511–4.760)*
Low birth weight, n (%)	4(6.2)	17(3.5)	15(6.9)
Unadjusted OR (95%CI)	1.800(0.586–5.527)	1(ref)	2.405(1.002–4.177)*
Adjusted OR^c^ (95%CI)	1.744(0.195–15.563)	1(ref)	1.015(0.278–3.705)
Any pregnancy complication, n (%)	33(50.8)	274(57)	164(75.2)
Unadjusted OR (95%CI)	0.779(0.464–1.309)	1(ref)	2.294(1.606–3.277)**
Adjusted OR^a^ (95%CI)	0.764(0.435–1.341)	1(ref)	2.766(1.840–4.158)**

Abbreviations: ppBMI: pre-pregnancy body mass index; GDM: gestational diabetes mellitus; FBG: fasting blood glucose; PIH: pregnancy-induced hypertension; CS: cesarean delivery; LGA: large-for-gestational age; SGA: small for-gestational age; OR: odds ratio; 95%CI: 95% confidence interval

Adjusted OR: adjusted for maternal age, primiparity, gestational weeks, GWG, FBG and implementation on doctors’ advices

Adjusted OR^a^: adjusted for maternal age, primiparity, GWG, FBG and implementation on doctor’s advice

Adjusted OR^b^: adjusted for maternal age, primiparity, GWG, FBG, implementation on doctor’s advice, PIH and preeclampsia

Adjusted OR^c^: adjusted for maternal age, gestational weeks, primiparity, GWG, FBG, implementation on doctor’s advice, PIH and preeclampsia

*p value < 0.05; **p value < 0.001

### Maternal GWG and risks of adverse pregnancy outcomes in GDM women

Table 3 displays the impacts of different GWG ranges on adverse pregnancy outcomes in GDM women. Compared with being adequate GWG, being iGWG had a lower risk of any pregnancy complication (aOR 0.628, 95%CI 0.435–0.907) and being EGWG had a higher risk of any pregnancy complication (aOR 1.548, 95%CI 1.006–2.382) even after adjustment for confounders. Pregnancies with iGWG has also dramatically lower risks of PIH (aOR 0.215, 95%CI 0.055–0.835), CS (aOR 0.612, 95%CI 0.421–0.889), but higher risk of preterm birth (aOR 2.261, 95%CI 1.089–4.692). Moreover, it was found that pregnancies with EGWG faced prominently elevated risks of LGA (aOR 1.929, 95%CI 1.272–2.923) and macrosomia (aOR 2.753, 95%CI 1.519–4.989).

**Table 3:** Maternal GWG and risks of adverse pregnancy outcomes in GDM women

Outcomes	iGWG	Adequate GWG	EGWG
PIH, n (%)	5(2.1)	18(5.5)	22(10.1)
Unadjusted OR (95%CI)	0.372(0.136–1.018)	1(ref)	1.952(1.006–3.789)*
Adjusted OR (95%CI)	0.215(0.055–0.835)*	1(ref)	1.669(0.801–3.476)
Preeclampsia, n (%)	2(0.8)	13(4)	9(4.6)
Unadjusted OR (95%CI)	0.206(0.046–0.922)*	1(ref)	1.164(0.488–2.774)
Adjusted OR (95%CI)	0.187(0.035–1.007)	1(ref)	1.051(0.408–2.708)
CS, n (%)	87(36.6)	164(49.8)	113(57.4)
Unadjusted OR (95%CI)	0.580(0.412–0.815)*	1(ref)	1.353(0.948–1.931)
Adjusted OR (95%CI)	0.612(0.421–0.889)*	1(ref)	1.279(0.860–1.903)
Preterm delivery, n (%)	24(10.1)	20(6.1)	11(5.6)
Unadjusted OR (95%CI)	1.749(0.942–3.247)	1(ref)	0.914(0.428–1.950)
Adjusted OR^a^ (95%CI)	2.261(1.089–4.692)*	1(ref)	1.215(0.520–2.838)
LGA, n (%)	39(16.4)	79(24)	76(38.6)
Unadjusted OR (95%CI)	0.620(0.405–0.950)*	1(ref)	1.988(1.356–2.914)*
Adjusted OR^b^ (95%CI)	0.657(0.421–1.026)	1(ref)	1.929(1.272–2.923)*
SGA, n (%)	3(1.3)	5(1.5)	4(2)
Unadjusted OR (95%CI)	0.827(0.196–3.496)	1(ref)	1.343(0.356–5.062)
Adjusted OR^b^ (95%CI)	0.680(0.120–3.845)	1(ref)	2.190(0.508–9.440)
Macrosomia, n (%)	10(4.2)	26(7.9)	36(18.3)
Unadjusted OR (95%CI)	0.524(0.248–1.109)	1(ref)	2.621(1.528–4.497)*
Adjusted OR^c^ (95%CI)	0.551(0.246–1.234)	1(ref)	2.753(1.519–4.989)*
Low birth weight, n (%)	18(7.6)	13(4)	5(2.5)
Unadjusted OR (95%CI)	2.041(0.979–4.254)	1(ref)	0.636(0.223–1.811)
Adjusted OR^c^ (95%CI)	1.863(0.500-6.937)	1(ref)	1.606(0.362–7.126)
Any pregnancy complication, n (%)	122(51.3)	208(63.2)	141(71.6)
Unadjusted OR (95%CI)	0.612(0.436–0.859)*	1(ref)	1.465(0.999–2.146)
Adjusted OR^a^ (95%CI)	0.628(0.435–0.907)*	1(ref)	1.548(1.006–2.382)*

Abbreviations: GDM: gestational diabetes mellitus; GWG: gestational weight gain; ppBMI: pre-pregnancy body mass index; iGWG: inadequate GWG; EGWG: excessive GWG; PIH: pregnancy-induced hypertension; CS: cesarean delivery; LGA: large-for-gestational age; SGA: small for-gestational age; FBG: fasting blood glucose; OR: odds ratio; 95%CI: 95% confidence interval

Adjusted OR: adjusted for maternal age, primiparity, gestational weeks, ppBMI, FBG and implementation on doctors’ advices

Adjusted OR^a^: adjusted for maternal age, primiparity, ppBMI, FBG and implementation on doctor’s advice

Adjusted OR^b^: adjusted for maternal age, primiparity, ppBMI, FBG, implementation on doctor’s advice, PIH and preeclampsia

Adjusted OR^c^: adjusted for maternal age, gestational weeks, primiparity, ppBMI, FBG, implementation on doctor’s advice, PIH and preeclampsia

*p value < 0.05; **p value < 0.001

### Maternal ppBMI and GWG status and risks of adverse pregnancy outcomes in GDM women

Table 4 presents subgroup analysis of the associations of the various GWG ranges with pregnancy complication in maternal pre-pregnancy normal and overweight/obese categories. Taken normal weight mothers with adequate GWG as reference, normal weight mothers with EGWG, overweight/obese mothers with adequate GWG and EGWG faced 1.745-, 2.356- and 3.064-fold risks of any pregnancy complication even in the adjusted models. Overweight/obese mothers with EGWG had the highest risks of PIH [aOR 3.113, 95% CI 1.257–7.709], CS (aOR 2.699, 95%CI 1.535–4.746), preterm delivery (aOR 5.810, 95%CI 1.745–19.342), LGA (aOR 2.892, 95%CI 1.185–3.695) and macrosomia (aOR 3.679, 95%CI 1.731–7.819). Interestingly, normal weight mothers with iGWG had obviously lower risks of PIH (aOR 0.086, 95%CI 0.009–0.812) and preeclampsia (aOR 0.094, 95%CI 0.009–0.999).

**Table 4:** Maternal ppBMI and GWG status and risks of adverse pregnancy outcomes in GDM women

Outcomes	Normal weight				Overweight/obese		
	iGWG (n = 175)	Adequate GWG (n = 208)	EGWG (n = 98)		iGWG (n = 34)	Adequate GWG (n = 92)	EGWG (n = 92)
PIH, n (%)	2(1.1)	11(5.3)	6(6.1)		2(5.9)	6(6.5)	14(15.2)
Unadjusted OR (95%CI)	0.208(0.046–0.953)*	1(ref)	1.168(0.419–3.255)		1.119(0.237–5.286)	1.249(0.448–3.488)	3.214(1.399–7.387)*
Adjusted OR (95%CI)	0.086(0.009–0.812)*	1(ref)	1.437(0.496–4.160)		0.444(0.038–5.202)	1.041(0.337–3.214)	3.113(1.257–7.709)*
Preeclampsia, n (%)	1(0.6)	9(4.3)	3(3.1)		0(0)	4(4.3)	6(6.5)
Unadjusted OR (95%CI)	0.127(0.016–1.013)	1(ref)	0.698(0.185–2.638)		-	1.005(0.301–3.351)	1.543(0.533–4.468)
Adjusted OR (95%CI)	0.094(0.009–0.999)*	1(ref)	0.847(0.215–3.343)		-	0.668(0.169–2.641)	1.572(0.493–5.005)
CS, n (%)	66(37.7)	96(46.2)	48(49)		15(44.1)	55(59.8)	61(66.3)
Unadjusted OR (95%CI)	0.706(0.469–1.064)	1(ref)	1.120(0.692–1.811)		0.921(0.444–1.911)	1.734(1.054–2.853)*	2.296(1.377–3.827)*
Adjusted OR (95%CI)	1.054(1.000-1.111)	1(ref)	1.244(0.737-2.100)		1.167(0.503–2.707)	1.829(1.062–3.150)*	2.699(1.535–4.746)*
Preterm delivery, n (%)	16(9.1)	7(3.4)	2(2)		5(14.7)	12(13)	9(9.8)
Unadjusted OR (95%CI)	2.926(1.175–7.287)*	1(ref)	0.598(0.122–2.934)		4.951(1.474–16.633)	4.307(1.637–11.333)*	3.114(1.122–8.637)*
Adjusted OR^a^ (95%CI)	3.042(1.037–8.919)*	1(ref)	0.798(0.146–4.349)		5.116(1.474–17.757)*	5.570(1.662–18.662)*	5.810(1.745–19.342)*
LGA, n (%)	28(16)	47(22.6)	38(38.8)		8(23.5)	27(29.3)	35(38)
Unadjusted OR (95%CI)	0.652(0.388–1.096)	1(ref)	2.170(1.289–3.650)		1.054(0.448–2.482)	1.423(0.818–2.476)	2.103(1.236–3.580)*
Adjusted OR^b^ (95%CI)	0.668(0.390–1.147)	1(ref)	2.399(1.366–4.214)*		0.983(0.372–2.598)	1.128(0.621–2.049)	2.892(1.185–3.695)*
SGA, n (%)	2(1.1)	4(1.9)	1(1)		1(2.9)	0(0)	3(3.3)
Unadjusted OR (95%CI)	0.590(0.107–3.258)	1(ref)	0.526(0.058–4.767)		1.545(0.168–14.277)	-	1.719(0.377–7.841)
Adjusted OR^b^ (95%CI)	0.415(0.061–2.806)	1(ref)	0.646(0.068–6.130)		0.842(0.052–13.708)	-	1.870(0.396–8.837)
Macrosomia, n (%)	7(4)	16(7.7)	16(16.3)		3(8.8)	10(10.9)	19(20.7)
Unadjusted OR (95%CI)	0.515(0.207–1.283)	1(ref)	2.341(1.118–4.906)*		1.200(0.330–4.367)	1.500(0.653–3.447)	3.211(1.565–6.588)*
Adjusted OR^c^ (95%CI)	0292(0.032–2.710)	1(ref)	2.249(1.016–4.976)*		0.976(0.232–4.106)	1.047(0.420–2.612)	3.679(1.731–7.819)*
Low birth weight, n (%)	11(6.3)	5(2.4)	1(1)		4(11.8)	7(7.6)	4(4.3)
Unadjusted OR (95%CI)	2.809(0.956–8.249)	1(ref)	0.419(0.048–3.632)		5.600(1.421–22.063)	3.424(1.057–11.096)*	1.888(0.495–7.203)
Adjusted OR^c^ (95%CI)	1.012(0.149–6.862)	1(ref)	0.645(0.044–9.394)		3.656(0.206–64.757)	0.095(0.002–5.469)	1.926(0.285–13.029)
Any pregnancy complication, n (%)	88(50.3)	121(58.2)	65(66.3)		22(64.7)	71(77.2)	71(77.2)
Unadjusted OR (95%CI)	0.727(0.485–1.090)	1(ref)	1.416(0.858–2.338)		1.318(0.619–2.806)	2.431(1.3894.253)*	2.431(1.3894.253)*
Adjusted OR^a^ (95%CI)	0.703(0.458–1.080)	1(ref)	1.745(1.008–3.022)*		1.651(0.683–3.987)	2.356(1.294–4.288)*	3.064(1.636–5.739)**

Abbreviations: GDM: gestational diabetes mellitus; GWG: gestational weight gain; ppBMI: pre-pregnancy body mass index; iGWG: inadequate GWG; EGWG: excessive GWG; PIH: pregnancy-induced hypertension; CS: cesarean delivery; LGA: large-for-gestational age; SGA: small for-gestational age; FBG: fasting blood glucose; OR: odds ratio; 95%CI: 95% confidence interval

Adjusted OR: adjusted for maternal age, primiparity, gestational weeks, FBG and implementation on doctors’ advices

Adjusted OR^a^: adjusted for maternal age, primiparity, FBG and implementation on doctor’s advice

Adjusted OR^b^: adjusted for maternal age, primiparity, FBG, implementation on doctor’s advice, PIH and preeclampsia

Adjusted OR^c^: adjusted for maternal age, gestational weeks, primiparity, FBG, implementation on doctor’s advice, PIH and preeclampsia

*p value < 0.05; **p value < 0.001

## Discussion

In the present study, we found that maternal pre-pregnancy overweight/obesity and GWG were associated with adverse pregnancy outcomes in the already high-risk settings of GDM. Women with the combination of overweight/obesity and EGWG may confer the greatest risk of adverse outcomes even after adjustment for confounders.

Although majority of studies have investigated the impacts of maternal ppBMI on adverse outcomes including GDM women [34–36], few data addressed the relations of maternal ppBMI with perinatal outcomes in GDM women. Recently, a retrospective cohort study of 1263 GDM mother-child pairs indicated that maternal pre-pregnancy overweight and obesity were related to rising risks of LGA [1.87 (95%CI 1.37–2.55), 2.98 (95%CI 1.89–4.69)] and macrosomia [2.06 (95%CI 1.50–2.84), 2.89 (95%CI 1.78–4.70)] in comparison of maternal pre-pregnancy normal weight; however, this study collapsed maternal underweight into normal weight group given the rarity of underweight GDM women [37]. In our study, we stratified GDM women into underweight, normal weight and overweight/obese groups, and observed that 65 (8.5%) of 764 GDM women was underweight, similar as the figures of 108 (7.6%) and 96 (11.5%) underweight pregnancies in another two Chinese cohort studies of GDM women [34, 35]. Concurring with the previous reports [34, 35], our study revealed that the great odds of PIH, CS, preterm birth, LGA and macrosomia were found in women who were overweight/obese before pregnancy even after adjusting for possible confounding factors. Nonetheless, different from Miao et al. [34], we didn’t find reduced risks of LGA and macrosomia for underweight women in comparison with normal-weight women, which may stem from further adjustments for the blood glucose and lifestyle intervention in our study. Sun et al. [35] displayed that low weight birth, but not macrosomia, was likely to occur in underweight GDM women without considering other risk factors. Given above small samples of underweight in GDM women, further in-depth comparative studies are warranted to clarify the impacts of underweight on perinatal outcomes in GDM women.

Currently, GDM women often use the revised IOM’s guidelines to assess their GWG targets as no GDM-specific GWG guidelines are available. In our study, the rates of insufficient and excessive GWG in GDM women were 31.2% and 25.8%, similar as another Chinese study found that insufficient GWG and EGWG in GDM women were 33.9% and 31.2% [33], but lower than 38% of EGWG in another Australia study [38]. The lower rate of EGWG in our study may result from the fact that up to 76.2% GDM women in our study followed doctors’ advices, while diet or exercise wasn’t recorded in another Chinese study above [33] and nearly 50% of EGWG pregnancies were of European descent and Middle Eastern in the Australia study [38]. The studies on associations of adverse pregnant outcomes with GWG in GDM women mainly focused on EGWG. Both a multicenter randomized clinical trial and the ATLANTIC Gestational Diabetes Program showed that pregnancies with EGWG were more prone to CS, macrosomia and LGA [12, 39]; however, these two studies included the inadequate and adequate GWG into the non-excessive group as control. Whether insufficient and excessive GWG are modifiable risk factors for worse clinical outcomes in GDM women arouse more interests and it is also worth exploring. A recent retrospective cohort study of all GDM pregnancies demonstrated that the pregnancies with EGWG posed nearly a two-fold higher risk of LGA and macrosomia, and a 1.5-fold higher risk of CS as compared with those with appropriate GWG [38]. Shi and colleagues [33] also showed that GDM women with total GWG above IOM’s guidelines faced a 1.34-fold likelihood of CS, 1.55-fold likelihood of macrosomia and 2.82-fold likelihood of LGA in comparison to those with GWG within IOM’s guidelines. Consistent with these studies, our results also displayed that GDM mothers with EGWG bore 2.753-fold risk of macrosomia and 1.929-fold risk of LGA in comparison to those with appropriate GWG. Different from them, we didn’t observe an increased incidence of CS in pregnancies with EGWG. Interestingly, our subgroup analysis may help to explain the discrepancy in CS, where we found the odds of CS was significantly higher in overweight/obese mothers with adequate or EGWG, but not in normal weight mothers with EGWG when using women with normal pre-gestational weight and proper GWG as the reference group. Moreover, our subgroup analysis also helped to explain the contrary results regarding the effects of EGWG on hypertensive events in GDM women. Studies from Shi et al. [33] and Macrì et al. [40] demonstrated that GDM women with EGWG were at elevated risk of hypertensive disorders of pregnancy in relative to those within adequate GWG. Conversely, Gou et al. [41], Miao et al. [34] and our present study did not observe an association between gestational hypertension and EGWG in GDM women. This may be explained by our subgroup analyses, which we found that GDM women with both overweight/obesity and EGWG, but not normal weight with EGWG, exhibited a greater risk of developing PIH as compared to those with normal weight and adequate GWG.

Several previous studies have been made regarding the relations of inadequate GWG and perinatal outcomes in women with GDM, but with conflicting conclusions. For example, Shi and colleagues [33] showed that total GWG below IOM’s guidelines was related to an elevated risk of preterm birth, SGA and reduced odds of macrosomia and LGA. Gou et al. [41] and our results displayed that insufficient GWG dramatically increased the risk for preterm birth, but was not related to SGA, macrosomia and LGA. However, Scifres et al. [42] showed that both EGWG and insufficient GWG added the risk of macrosomia. Different regions, sample sizes, dietary cultures and characteristics of the study population may lead to these inconsistent conclusions. Furthermore, we observed that insufficient GWG could protect against CS, PIH and any pregnancy complication, contrary to previous reported results [43]. Align with us, a Brazilian study of 2244 GDM pregnant women concluded that women with less than the recommended GWG in the second trimester faced a lower risk of CS than those with sufficient GWG during that period [44]. According to these findings, we speculate that weight gain less than the revised IOM’s recommended weight gain may be adequate for women with GDM, but further large samples of prospective cohorts are needed to further verify these findings.

PpBMI reflects the nutritional conditions of pregnant women before conception, while GWG stands for the nutritional status of pregnant women during pregnancy [45, 46]. Pre-pregnancy overweight/obesity and EGWG as determinant factors of pregnancy outcomes are arousing more interests because it comes to the well-beings of both mothers and their babies [1, 2, 47]. Robust data revealed that maternal pre-pregnancy overweight/obesity and EGWG were highly related to maternal-infant adverse outcomes including women with GDM [47, 48], and more interests were generated to wonder which condition was worse by comparing overweight/obese alone with EGWG alone in women with GDM. Recently, several population-based cohort studies documented the pre-pregnancy obesity had a greater impact on adverse pregnancy outcomes than EGWG; however, those conclusions were indirectly obtained through numeric comparison on the risks of adverse perinatal outcomes after comparing obesity with normal ppBMI and contrasting EGWG with adequate GWG, respectively [8, 49]. By contrast, Gujski and his colleagues showed that weaker associations of mothers with pre-gestational obesity and perinatal outcomes were observed as compared to mothers with EGWG in diet controlled GDM women without considering risk factors such as maternal age [50]. The above inconsistent conclusions may be partially explained by our study that we observed that overweight/obese mothers with adequate GWG (overweight/obese alone) had stronger associations with maternal outcomes than EGWG mothers with normal weight (EGWG alone), while EGWG alone exhibited higher risk of neonatal outcomes than overweight/obese alone. Our findings indicated that maternal overweight/obesity tended to maternal complications and EGWG related to fetal complications. Of note, we found the combination of overweight/obese and EGWG had a greater risk for any pregnancy complication than either overweight/obesity or EGWG alone. These findings suggested that it was necessary to make preconception assessments and counselling for overweight/obese women to obtain good pregnancy outcomes by losing weight before pregnancy and controlling weight gain throughout pregnancy.

Several limitations should be considered in our present study. Firstly, our study participants were Chinese Han pregnant women and more studies may need to generalize our findings to other races or nationalities. Secondly, we used the recorded implementation of doctors’ advices on lifestyle changes including diet modification, self-monitor glucose and exercise for every GDM women, instead of the incomplete data like no demographic characteristics such as education, occupation and lifestyle factors such as smoking and drinking during pregnancy as well as physical activity. In our previous study [21], we have observed that inpatients with GDM in the implementation group had lower frequencies of PIH, preeclampsia and macrosomia than those in the non-implementation group, which indicated that there was a modest benefit for pregnancy outcomes to treatment for GDM. Therefore, in our opinion, whether 80% of doctors’ advices were implemented or not has already covered almost the majority of maternal demographic characteristics and lifestyle factors and we also included the implementation of doctors’ advices into the multiple regression models. Thirdly, given the relatively small sample size in the underweight group, the subgroup analysis regarding the associations of the various GWG ranges with pregnancy complication were not conducted in the underweight group, which was also supported by the fact that underweight women with GDM were quite few according to previously reported studies [34, 35]. Fourthly, the causality of maternal ppBMI and GWG with adverse pregnancy outcomes could not be established as the nature of retrospective study, further large prospective studies may need to clarify our current findings.

## Conclusions

Our study revealed that maternal pre-pregnancy overweight/obesity and GWG were associated with adverse pregnancy outcomes and women with the combination of overweight/obesity and EGWG may confer the greatest risk of adverse outcomes in the already high-risk settings of GDM. Our study indicated that clinicians should urge overweight/obese women to lose weight when planning pregnancy and supervise them to control weight gain during pregnancy to minimize adverse obstetric outcomes. Further prospective studies are needed to determine whether narrower GWG targets would provide additional safety benefits in this population.

## Data Availability

The datasets used and/or analysed during the current study are available from the corresponding authors on reasonable request.

## Abbreviations

95%CI: 95% confidence interval
aOR: Adjusted odds ratio
CS: Cesarean delivery
EGWG: Excessive GWG
FBG: Fasting blood glucose
GDM: Gestational diabetes mellitus
GWG: Gestational weight gain
IADPSG: International Association of Diabetes and Pregnancy Study Groups
iGWG: Inadequate GWG
IOM: Institute of Medicine
LGA: Large-for-gestational age
OGTT: Oral glucose tolerance test
PIH: Pregnancy-induced hypertension
ppBMI: pre-pregnancy body mass index
SGA: Small for-gestational age

## References

[1] Mastroeni MF, Czarnobay SA, Kroll C, Figueirêdo KB, Mastroeni SS, Silva JC (2017). The Independent Importance of Pre-pregnancy Weight and Gestational Weight Gain for the Prevention of large-for gestational age brazilian newborns. Matern Child Health J.

[2] Li C, Liu Y, Zhang W (2015). Joint and Independent Associations of Gestational Weight Gain and Pre-Pregnancy Body Mass Index with Outcomes of pregnancy in Chinese Women: a retrospective cohort study. PLoS ONE.

[3] Li N, Liu E, Guo J, Pan L, Li B, Wang P (2013). Maternal prepregnancy body mass index and gestational weight gain on pregnancy outcomes. PLoS ONE.

[4] Chen CN, Chen HS, Hsu HC (2020). Maternal prepregnancy body Mass Index, Gestational Weight Gain, and risk of adverse perinatal outcomes in Taiwan: a Population-Based birth cohort study. Int J Environ Res Public Health.

[5] Tang J, Zhu X, Chen Y, Huang D, Tiemeier H, Chen R (2021). Association of maternal pre-pregnancy low or increased body mass index with adverse pregnancy outcomes. Sci Rep.

[6] Santos S, Voerman E, Amiano P, Barros H, Beilin LJ, Bergström A (2019). Impact of maternal body mass index and gestational weight gain on pregnancy complications: an individual participant data meta-analysis of european, north american and australian cohorts. BJOG.

[7] Dude AM, Grobman W, Haas D, Mercer BM, Parry S, Silver RM (2021). Gestational weight gain and pregnancy outcomes among Nulliparous Women. Am J Perinatol.

[8] Fayed A, Wahabi HA, Esmaeil S, Elkouny R, Elmorshedy H, Bakhsh H (2022). Independent effect of gestational weight gain and prepregnancy obesity on pregnancy outcomes among saudi women: a sub-cohort analysis from Riyadh mother and baby cohort study (RAHMA). PLoS ONE.

[9] Sun Y, Shen Z, Zhan Y, Wang Y, Ma S, Zhang S (2020). Effects of pre-pregnancy body mass index and gestational weight gain on maternal and infant complications. BMC Pregnancy Childbirth.

[10] Bodnar LM, Siega-Riz AM, Simhan HN, Himes KP, Abrams B (2010). Severe obesity, gestational weight gain, and adverse birth outcomes. Am J Clin Nutr.

[11] Institute of Medicine (US) Committee on Nutritional Status During Pregnancy and Lactation (1990). Nutrition during pregnancy: Part I Weight Gain: part II nutrient supplements.

[12] Blackwell SC, Landon MB, Mele L, Reddy UM, Casey BM, Wapner RJ (2016). Relationship between excessive gestational weight gain and neonatal adiposity in women with mild gestational diabetes mellitus. Obstet Gynecol.

[13] Langford A, Joshu C, Chang JJ, Myles T, Leet T (2011). Does gestational weight gain affect the risk of adverse maternal and infant outcomes in overweight women?. Matern Child Health J.

[14] Abenhaim HA, Kinch RA, Morin L, Benjamin A, Usher R (2007). Effect of prepregnancy body mass index categories on obstetrical and neonatal outcomes. Arch Gynecol Obstet.

[15] Ferrari N, Mallmann P, Brockmeier K, Strüder HK, Graf C (2014). Secular trends in pregnancy weight gain in german women and their influences on foetal outcome: a hospital-based study. BMC Pregnancy Childbirth.

[16] Flegal KM, Carroll MD, Ogden CL, Curtin LR (2010). Prevalence and trends in obesity among US adults, 1999–2008. JAMA.

[17] Liu Y, Dai W, Dai X, Li Z (2012). Prepregnancy body mass index and gestational weight gain with the outcome of pregnancy: a 13-year study of 292,568 cases in China. Arch Gynecol Obstet.

[18] Ma GS, Li YP, Wu YF, Zhai FY, Cui ZH, Hu XQ (2005). The prevalence of body overweight and obesity and its changes among chinese people during 1992 to 2002. Zhonghua Yu Fang Yi Xue Za Zhi.

[19] Kominiarek MA, Peaceman AM (2017). Gestational weight gain. Am J Obstet Gynecol.

[20] Kominiarek MA, Saade G, Mele L, Bailit J, Reddy UM, Wapner RJ (2018). Association between Gestational Weight Gain and Perinatal Outcomes. Obstet Gynecol.

[21] Li MF, Ma L, Yu TP, Zhu Y, Chen MY, Liu Y (2020). Adverse maternal and neonatal outcomes in pregnant women with abnormal glucose metabolism. Diabetes Res Clin Pract.

[22] Narayan KM, Boyle JP, Thompson TJ, Gregg EW, Williamson DF (2007). Effect of BMI on lifetime risk for diabetes in the U.S. Diabetes Care.

[23] Hedderson MM, Gunderson EP, Ferrara A (2010). Gestational weight gain and risk of gestational diabetes mellitus. Obstet Gynecol.

[24] Yao D, Chang Q, Wu QJ, Gao SY, Zhao H, Liu YS (2020). Relationship between maternal central obesity and the risk of gestational diabetes Mellitus: a systematic review and Meta-analysis of Cohort Studies. J Diabetes Res.

[25] Kim SY, England L, Wilson HG, Bish C, Satten GA, Dietz P (2010). Percentage of gestational diabetes mellitus attributable to overweight and obesity. Am J Public Health.

[26] Li MF, Ke JF, Ma L, Wang JW, Zhang ZH, Li JB (2022). Maternal pre-pregnancy obesity combined with abnormal glucose metabolism further increases adverse pregnancy outcomes in chinese pregnant women. Front Endocrinol (Lausanne).

[27] Li MF, Ma L, Feng QM, Zhu Y, Yu TP, Ke JF (2020). Effects of maternal subclinical hypothyroidism in early pregnancy diagnosed by different criteria on adverse perinatal outcomes in chinese women with negative TPOAb. Front Endocrinol (Lausanne).

[28] Metzger BE, Gabbe SG, Persson B, Buchanan TA, Catalano PA, International Association of Diabetes and Pregnancy Study Groups Consensus Panel (2010). International association of diabetes and pregnancy study groups recommendations on the diagnosis and classification of hyperglycemia in pregnancy. Diabetes Care.

[29] American Diabetes Association (2014). Standards of medical care in diabetes–2014. Diabetes Care.

[30] He W, Li Q, Yang M, Jiao J, Ma X, Zhou Y (2015). Lower BMI cutoffs to define overweight and obesity in China. Obes (Silver Spring).

[31] Organization WH. International statistical classification of diseases and related health problems. 10th revision. 2nd ed. World Health Organization; 2004.

[32] Zhu L, Zhang R, Zhang S, Shi W, Yan W, Wang X (2015). Chinese neonatal birth weight curve for different gestational age. Zhonghua Er Ke Za Zhi.

[33] Shi P, Liu A, Yin X (2021). Association between gestational weight gain in women with gestational diabetes mellitus and adverse pregnancy outcomes: a retrospective cohort study. BMC Pregnancy Childbirth.

[34] Miao M, Dai M, Zhang Y, Sun F, Guo X, Sun G (2017). Influence of maternal overweight, obesity and gestational weight gain on the perinatal outcomes in women with gestational diabetes mellitus. Sci Rep.

[35] Sun D, Li F, Zhang Y, Xu X (2014). Associations of the pre-pregnancy BMI and gestational BMI gain with pregnancy outcomes in chinese women with gestational diabetes mellitus. Int J Clin Exp Med.

[36] Zheng QX, Wang HW, Jiang XM, Lin Y, Liu GH, Pan M (2022). Prepregnancy body mass index and gestational weight gain are associated with maternal and infant adverse outcomes in chinese women with gestational diabetes. Sci Rep.

[37] Leng J, Li W, Zhang S, Liu H, Wang L, Liu G (2015). GDM Women’s Pre-Pregnancy Overweight/Obesity and gestational weight gain on offspring overweight status. PLoS ONE.

[38] Wong T, Barnes RA, Ross GP, Cheung NW, Flack JR (2017). Are the Institute of Medicine weight gain targets applicable in women with gestational diabetes. mellitus? Diabetologia.

[39] Egan AM, Dennedy MC, Al-Ramli W, Heerey A, Avalos G, Dunne F (2014). ATLANTIC-DIP: excessive gestational weight gain and pregnancy outcomes in women with gestational or pregestational diabetes mellitus. J Clin Endocrinol Metab.

[40] Macrì F, Pitocco D, di Pasquo E, Salvi S, Rizzi A, Di Leo M (2018). Gestational weight gain as an independent risk factor for adverse pregnancy outcomes in women with gestational diabetes. Eur Rev Med Pharmacol Sci.

[41] Gou BH, Guan HM, Bi YX, Ding BJ (2019). Gestational diabetes: weight gain during pregnancy and its relationship to pregnancy outcomes. Chin Med J (Engl).

[42] Leunissen RW, Kerkhof GF, Stijnen T, Hokken-Koelega A (2009). Timing and tempo of first-year rapid growth in relation to cardiovascular and metabtabolic risk profile in early adulthood. JAMA.

[43] Margerison Zilko CE, Rehkopf D, Abrams B (2010). Association of maternal gestational weight gain with short- and long-term maternal and child health outcomes. Am J Obstet Gynecol.

[44] Drehmer M, Duncan BB, Kac G, Schmidt MI (2013). Association of second and third trimester weight gain in pregnancy with maternal and fetal outcomes. PLoS ONE.

[45] Alberico S, Montico M, Barresi V, Monasta L, Businelli C, Soini V (2014). The role of gestational diabetes, pre-pregnancy body mass index and gestational weight gain on the risk of newborn macrosomia: results from a prospective multicentre study. BMC Pregnancy Childbirth.

[46] Gaillard R (2015). Maternal obesity during pregnancy and cardiovascular development and disease in the offspring. Eur J Epidemiol.

[47] Voerman E, Santos S, Inskip H, Amiano P, Barros H, LifeCycle Project-Maternal Obesity and Childhood Outcomes Study Group (2019). Association of gestational weight gain with adverse maternal and infant outcomes. JAMA.

[48] Badon SE, Dublin S, Nance N, Hedderson MM, Neugebauer R, Easterling T (2021). Gestational weight gain and adverse pregnancy outcomes by pre-pregnancy BMI category in women with chronic hypertension: a cohort study. Pregnancy Hypertens.

[49] Gaillard R, Durmuş B, Hofman A, Mackenbach JP, Steegers EA, Jaddoe VW (2013). Risk factors and outcomes of maternal obesity and excessive weight gain during pregnancy. Obes (Silver Spring).

[50] Gujski M, Szukiewicz D, Chołuj M, Sawicki W, Bojar I (2020). Fetal and placental weight in pre-gestational maternal obesity (PGMO) vs. excessive gestational weight gain (EGWG)-a preliminary approach to the perinatal outcomes in diet-controlled gestational diabetes mellitus. J Clin Med.

